# Validity of influenza vaccine effectiveness calculations using a test-negative design in Thailand

**DOI:** 10.1093/aje/kwag094

**Published:** 2026-04-29

**Authors:** Erica E Zeno, Prabda Praphasiri, Wichan Bhunyakitikorn, Sutthichai Nakphook, Darunee Ditsungnoen, Wuttiphong Phakdeekul, Passakorn Ongarj, Ratchadaporn Ungcharoen, Warinmad Kedthongma, Seesai Yeesoonsang, Kanlaya Sornwong, Chaninan Sonthichai, Anna N Chard, William W Davis, Martha P Montgomery, Kriengkrai Prasert

**Affiliations:** Influenza Division, National Center for Immunization and Respiratory Diseases, US Centers for Disease Control and Prevention, Atlanta, GA, United States; Epidemic Intelligence Service, US Centers for Disease Control and Prevention, Atlanta, GA, United States; Thailand Ministry of Public Health-US Centers for Disease Control and Prevention Collaboration, Nonthaburi, Thailand; Faculty of Public Health, Kasetsart University Chalermphrakiat Sakon Nakhon Province Campus, Sakon Nakhon Province, Thailand; Division of Epidemiology, Department of Disease Control, Ministry of Public Health, Nonthaburi, Thailand; Faculty of Public Health, Kasetsart University Chalermphrakiat Sakon Nakhon Province Campus, Sakon Nakhon Province, Thailand; Nakhon Phanom Provincial Hospital, Nakhon Phanom, Thailand; Thailand Ministry of Public Health-US Centers for Disease Control and Prevention Collaboration, Nonthaburi, Thailand; Faculty of Public Health, Kasetsart University Chalermphrakiat Sakon Nakhon Province Campus, Sakon Nakhon Province, Thailand; Faculty of Public Health, Kasetsart University Chalermphrakiat Sakon Nakhon Province Campus, Sakon Nakhon Province, Thailand; Faculty of Public Health, Kasetsart University Chalermphrakiat Sakon Nakhon Province Campus, Sakon Nakhon Province, Thailand; Faculty of Public Health, Kasetsart University Chalermphrakiat Sakon Nakhon Province Campus, Sakon Nakhon Province, Thailand; Division of Epidemiology, Department of Disease Control, Ministry of Public Health, Nonthaburi, Thailand; Nakhon Phanom Provincial Hospital, Nakhon Phanom, Thailand; Thailand Ministry of Public Health-US Centers for Disease Control and Prevention Collaboration, Nonthaburi, Thailand; Influenza Division, National Center for Immunization and Respiratory Diseases, US Centers for Disease Control and Prevention, Atlanta, GA, United States; Influenza Division, National Center for Immunization and Respiratory Diseases, US Centers for Disease Control and Prevention, Atlanta, GA, United States; Thailand Ministry of Public Health-US Centers for Disease Control and Prevention Collaboration, Nonthaburi, Thailand; Influenza Division, National Center for Immunization and Respiratory Diseases, US Centers for Disease Control and Prevention, Atlanta, GA, United States; Thailand Ministry of Public Health-US Centers for Disease Control and Prevention Collaboration, Nonthaburi, Thailand; Faculty of Public Health, Kasetsart University Chalermphrakiat Sakon Nakhon Province Campus, Sakon Nakhon Province, Thailand; Nakhon Phanom Provincial Hospital, Nakhon Phanom, Thailand

**Keywords:** vaccine effectiveness, influenza, test-negative design

## Abstract

Thailand uses a test-negative design to calculate influenza vaccine effectiveness (VE) annually for the Southern Hemisphere formulation and shares estimates with the World Health Organization to inform global vaccine strain selection. This study analyzed potential misclassification and confounding factors to evaluate validity of the test-negative design in Thailand. We retrospectively reviewed influenza sentinel surveillance data from June 2023 to May 2024 and gathered information on vaccination history and health-seeking behavior. Cases were medically attended illnesses with positive influenza test results; noncases had negative results for influenza and SARS-CoV-2. We compared VE using the routine approach with six scenarios, representing four potential threats to the validity of test-negative design studies. We assessed exposure (vaccination) misclassification, outcome (influenza) misclassification, and confounding by health-seeking behavior. Unmeasured confounding was evaluated by calculating VE against rhinovirus, an outcome unrelated to influenza vaccination. A total of 4709 patients were included, of which 328 (7.0%) were vaccinated against influenza. Under the routine approach, VE against medically attended influenza was 36.9% (95% CI, 13.5%-53.9%). In alternative scenarios, VE ranged from 32.4% to 42.8%, with overlapping 95% CIs. These findings support the validity of Thailand’s VE estimates and can inform improvements in surveillance procedures.

## Introduction

The test-negative design is widely used to calculate influenza vaccine effectiveness, though it relies on several assumptions that, if not considered, might lead to biased estimates. Vaccination is an important prevention strategy against influenza. Because of antigenic drift, the influenza vaccine is reformulated twice per year for Northern and Southern hemispheres. Vaccine effectiveness varies annually depending on the degree of antigenic match with circulating influenza virus strains and on dynamic host and environmental factors and thus needs to be recalculated for different seasons and in different populations.^1^ In Thailand, influenza vaccination recommendations and number of doses purchased has expanded over the last 2 decades.^2^^-^^4^ Information on vaccine effectiveness helps public health leaders in Thailand to decide whether to continue or expand influenza vaccination programs, and vaccine effectiveness results from Southern Hemisphere vaccine used in Thailand help to inform vaccine strain selection for the subsequent Northern Hemisphere influenza vaccine formulation.^5^

In 2023, Thailand’s Advisory Committee on Immunization Practices, which is not affiliated with the US Advisory Committee on Immunization Practices, recommended influenza vaccination for health care personnel, children aged 6-35 months, pregnant women, people with certain underlying medical conditions, adults aged 65 years and older, and people who work or live in prisons. Each year, the National Health Security Office (NHSO) purchases and administers approximately 4-5 million doses of Southern Hemisphere influenza vaccine, which covers approximately one-third of the recommended groups.^6^ Individuals can also receive influenza vaccination from private clinics, purchased through self-pay or by their employer. An additional 4-5 million doses of privately purchased influenza vaccine are administered in Thailand.^3^ Privately purchased vaccines are available around March each year, and publicly purchased vaccine is administered from May through August.

Thailand conducts sentinel surveillance to monitor influenza trends.^7^ Sentinel surveillance data are used to calculate influenza vaccine effectiveness using a test-negative design. Vaccine effectiveness estimates are shared with the World Health Organization Global Influenza Vaccine Effectiveness network twice a year to inform vaccine strain selection for northern and Southern Hemisphere vaccines.^7^^-^^9^ The test-negative design has been used in many countries to calculate influenza vaccine effectiveness for at least 20 years.^10^^-^^15^ It has been applied in outpatient and inpatient settings, but several assumptions should be considered in its application. For example, one advantage of the test-negative design is that it accounts for differences in health-seeking behavior between patients who are and are not vaccinated. However, residual confounding might exist if health-seeking behavior is incompletely captured as a binary variable.^16^ Other assumptions include accurate classification of vaccination status, accurate classification of influenza status, and minimization of selection bias and confounding.^11^^,^^13^

This analysis aimed to assess the validity of the test-negative design for calculating influenza vaccine effectiveness against medically attended illness within Thailand’s sentinel surveillance program by estimating the degree of misclassification and residual or unmeasured confounding in its current approach.

## Methods

We analyzed the validity of the test-negative design by analyzing data from Thailand’s influenza sentinel surveillance system. Thailand’s Department of Epidemiology conducted sentinel surveillance for influenza-like illness (ILI) and severe acute respiratory infection (SARI) in 8 hospital sites across Thailand. This analysis included sentinel surveillance data for all ILI and SARI surveillance cases from 6 sentinel sites from 1 influenza season (June 2023 through May 2024). Two sites were excluded because they focused on avian influenza surveillance. According to the World Health Organization, ILI is defined as measured fever of 38°C or more and cough with onset within the past 10 days. SARI is defined as reported or measured fever of 38°C or more, cough with onset within the past 10 days, and hospitalization.^17^ At each sentinel site, patients were screened by a trained surveillance officer at outpatient and inpatient locations. Nasopharyngeal or nasal swab specimens were collected from 4 patients per day. Two specimens were from ILI cases and 2 specimens were from SARI cases, 1 specimen each from a patient under 5 years of age and 1 from a patient 5 years of age or older. Each day, surveillance officers included the first patients who fit these criteria who agreed to enroll in surveillance. All specimens were tested by multiplex, reverse transcriptase polymerase chain reaction (rtPCR) for 23 respiratory pathogen targets, including influenza virus and SARS-CoV-2 using the QIAstat panel. Data on demographics, underlying medical conditions, influenza and COVID-19 vaccination history, clinical data, and recent exposures were collected for each patient on a standardized case report form. This activity was reviewed by CDC, deemed not research, and was conducted consistent with applicable federal law and CDC policy.

Thailand’s methods for calculating vaccine effectiveness have been described previously.^9^ Briefly, medically attended illnesses with positive test results for influenza were considered cases and illnesses with negative test results for influenza and SARS-CoV-2 were considered noncases. Illnesses with positive test results for SARS-CoV-2 were excluded.^18^ Patients were considered vaccinated against influenza if they received at least 1 dose of influenza vaccine during May 2023 through April 2024 (corresponding with availability of Southern Hemisphere vaccine) and at least 14 days prior to symptom onset. Vaccine effectiveness against medically attended influenza illness was calculated by comparing the odds of influenza vaccination between cases and noncases using multivariable logistic regression as (1 − adjusted odds ratio) × 100%. Odds ratios were adjusted for age (as a continuous variable), sex, calendar week of symptom onset, self-reported presence of 1 or more underlying condition, and receipt of 2 or more COVID-19 vaccine doses. This approach was considered the baseline scenario.

Using 6 different scenarios, we examined 4 potential sources of misclassification and bias—misclassification of the exposure (influenza vaccination status), misclassification of the outcome (lab-confirmed influenza), residual confounding by health-seeking behavior, and unmeasured confounding. To assess misclassification of influenza vaccination status (scenario 1), we compared vaccination history as recorded in the surveillance case report form with 2 or 3 additional data sources. One was the hospital electronic medical record, and the second was the Krung Thai Digital Health Platform, which is used nationally by hospitals for reimbursement of publicly purchased vaccines. For children, vaccinations could also be recorded in the mother’s pink book, which records medical visits and vaccinations from prenatal visits through age 5 years. According to standard procedure, history in the surveillance case report form is recorded based on a combination of self-report and documented verification. Each patient is asked about self-reported vaccination history. If the patient reports vaccination, the vaccination record is verified in the electronic medical record, the Krung Thai Digital Health Platform, or (for children) the pink book; however, in practice procedures vary slightly from site to site and it is unclear how much verification occurs. Additionally, because some patients may not recall receiving influenza vaccination, vaccinations in the surveillance case report form might be underrecorded. Reviewing vaccination documentation for all ILI and SARI cases in the electronic medical record, the Krung Thai Digital Health Platform, and (for children) the pink book might uncover more vaccinations.

To assess misclassification of lab-confirmed influenza status, we compared the current ILI and SARI case definitions with alternative definitions. Although the ILI and SARI definitions include patients with symptom onset in the last 10 days, viral shedding peaks around 2 days after exposure, prior to the onset of symptoms and often becomes undetectable 5 days after symptom onset.^19^^,^^20^ Therefore, some patients with influenza virus infection who are tested after 5 days might test negative and be misclassified as negative for influenza. In the alternative ILI and SARI definition (scenario 2), we excluded all patients with symptom onset more than 5 days prior to enrollment. Secondly, we compared an alternative definition for noncases because previous studies have noted the importance of appropriate selection of noncases.^21^^,^^22^ In the baseline scenario, influenza-like illnesses that test negative for influenza and negative for SARS-CoV-2 are included in the noncase group for comparison, including illnesses that are positive for another respiratory virus and those that are negative for all pathogens. In the alternative noncase definition (scenario 3), we included only illnesses that tested positive for a noninfluenza, non-SARS-CoV-2 virus.

To assess residual confounding bias by differences in health-seeking behavior, we extracted from the electronic medical record the number of health care visits (outpatient, inpatient, and emergency department visits) in the 12 months preceding enrollment in surveillance as a proxy for health-seeking behavior. We performed descriptive analyses to compare differences in health care visits by vaccination status and case status. In scenario 4, we adjusted for health care visits, categorized into quartiles, as a potential confounder in the multivariable model to calculate vaccine effectiveness. In scenario 5, we restricted the analysis to patients with at least 1 health care visit in the last year.

We assessed for unmeasured confounding by estimating influenza vaccine effectiveness against rhinovirus, an unrelated outcome (scenario 6).^23^ We selected rhinovirus as the outcome for several reasons: rhinovirus is not a vaccine-preventable disease, influenza vaccination is not expected to alter protection against or susceptibility to rhinovirus; rhinovirus rtPCR test results were available for all patients; and rhinovirus was commonly detected in the study population, providing sufficient sample size for analysis. Lastly, to assess the impact of multiple sources of bias, we combined and calculated VE for scenarios 1 and 2, and scenarios 1, 2, and 5.

## Results

During June 2023 through May 2024, there were 4709 patients with ILI or SARI enrolled from 6 surveillance sites. A total of 328 ILI/SARI patients were recorded as vaccinated against influenza in the surveillance case report form. After reviewing electronic medical records, the Krung Thai Digital Health Platform, and pink book records (for children) at all 6 sites, 60 unvaccinated patients were reclassified as vaccinated under scenario 1 (Table 1). Among 60 reclassified patients, 40 (67%) were children aged 3-17 years, 28 (47%) were female, 32 (53%) had preexisting conditions, and 24 (40%) were enrolled from an outpatient setting.

**Table 1:** Agreement among three vaccination record systems for influenza vaccination by site and demographic characteristics, Thailand, June 2023-May 2024.

	**Total *n* = 4709**	**Vaccinated according to surveillance site case report form, *n* = 328**	**Vaccinated according to any data system, *n* = 388**	**Reclassified patients, *n* = 60** ^**a**^
Surveillance site				
Mae Chan	981 (20.8%)	33 (10.1%)	33 (8.5%)	0
Nakhon Phanom	864 (18.3%)	7 (2.1%)	7 (1.8%)	0
Phra Nakhon Si Ayutthaya	620 (13.2%)	53 (16.2%)	63 (16.2%)	10 (16.7%)
Phrapokklao	678 (14.4%)	79 (24.1%)	89 (22.9%)	10 (16.7%)
Queen Sirikit National Institute of Child Health	920 (19.5%)	136 (41.5%)	174 (44.8%)	38 (63.3%)
Koh Samui	646 (13.7%)	20 (6.1%)	22 (5.7%)	2 (3.3%)
Age group				
0-35 mo	1725 (36.6%)	72 (22.0%)	87 (22.4%)	15 (25.0%)
3-17 y	2100 (44.6%)	107 (32.6%)	147 (37.9%)	40 (66.7%)
18-64 y	627 (13.3%)	110 (33.5%)	114 (29.4%)	4 (6.7%)
65 y and older	257 (5.5%)	39 (11.9%)	40 (10.3%)	1 (1.7%)
Sex				
Female	2132 (45.3%)	184 (56.1%)	212 (54.6%)	28 (46.7%)
Male	2577 (54.7%)	144 (43.9%)	176 (45.4%)	32 (53.3%)
Preexisting conditions				
Yes	926 (19.7%)	134 (40.9%)	166 (42.8%)	32 (53.3%)
No	3783 (80.3%)	194 (59.1%)	222 (57.2%)	28 (46.7%)
Clinical setting				
Outpatient	1939 (41.2%)	197 (60.1%)	221 (57.0%)	24 (40.0%)
Inpatient	2567 (54.5%)	121 (36.9%)	155 (39.9%)	34 (56.7%)
Intensive Care Unit	203 (4.3%)	10 (3.0%)	12 (3.1%)	2 (3.3%)

Some columns might not add to 100% due to rounding.

a Number of patients reclassified under scenario 1, assessing misclassification of the exposure.

**Figure 1:**
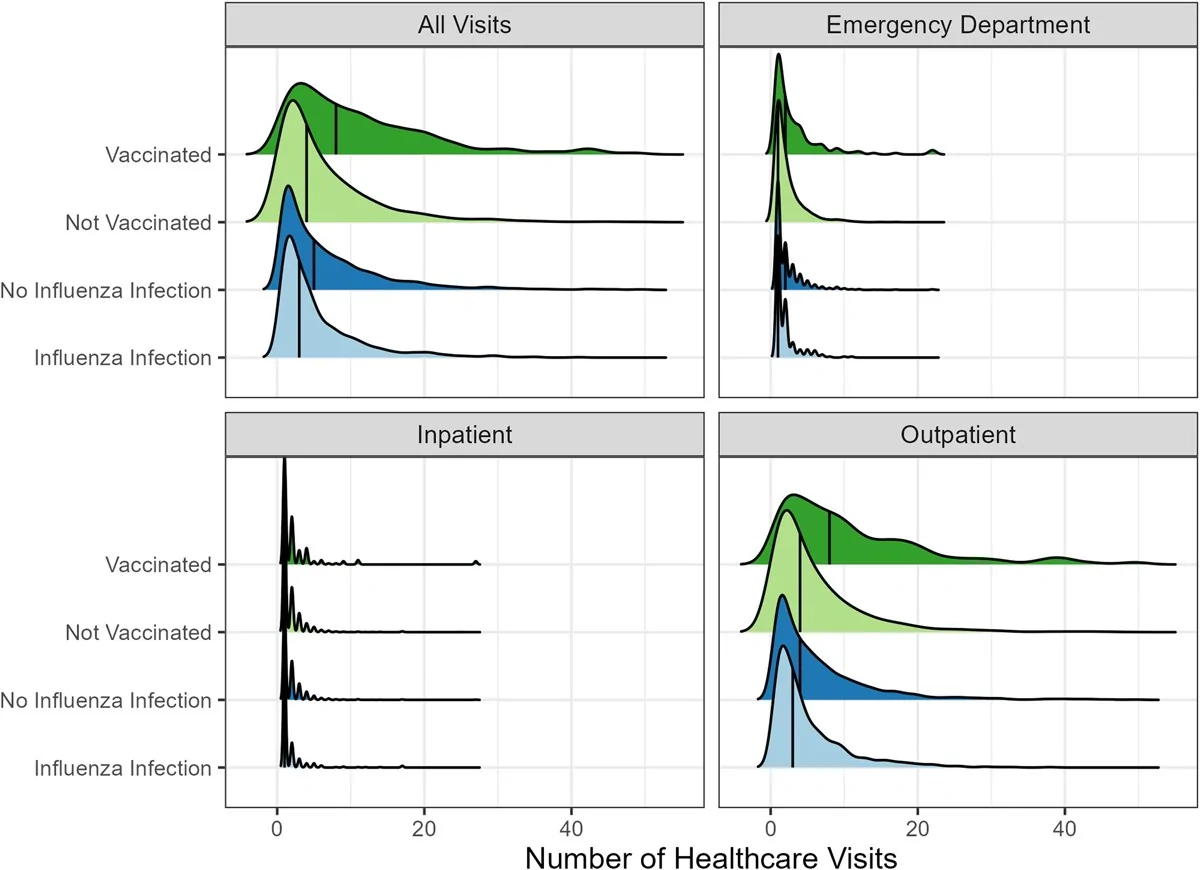
Differences in health care visits in the preceding 12 months by outcome (influenza) status and exposure (influenza vaccination) status, Thailand, June 2023-May 2024. Legend. Influenza vaccination status was defined according to original documentation in the surveillance case report form. No influenza infection included all illnesses that had negative rtPCR results for influenza, including SARS-CoV-2, other respiratory pathogens, and pan-negative illnesses. Patients with more than 50 visits for outpatient visits or all visits in the previous 12 months were truncated.

The distribution of the number of health care visits in the previous 12 months was right-skewed as some patients had over 100 recorded hospital visits (Figure 1 ). Patients with influenza had a lower median number of visits (3 visits) compared to patients without influenza (4 visits) (*P*-value < .001). Vaccinated patients had a higher median number of visits (8 visits) compared to unvaccinated patients (4 visits) (*P*-value < .001). We stratified the number of health care visits by setting (outpatient, inpatient, and emergency department) and found similar results.

**Figure 2:**
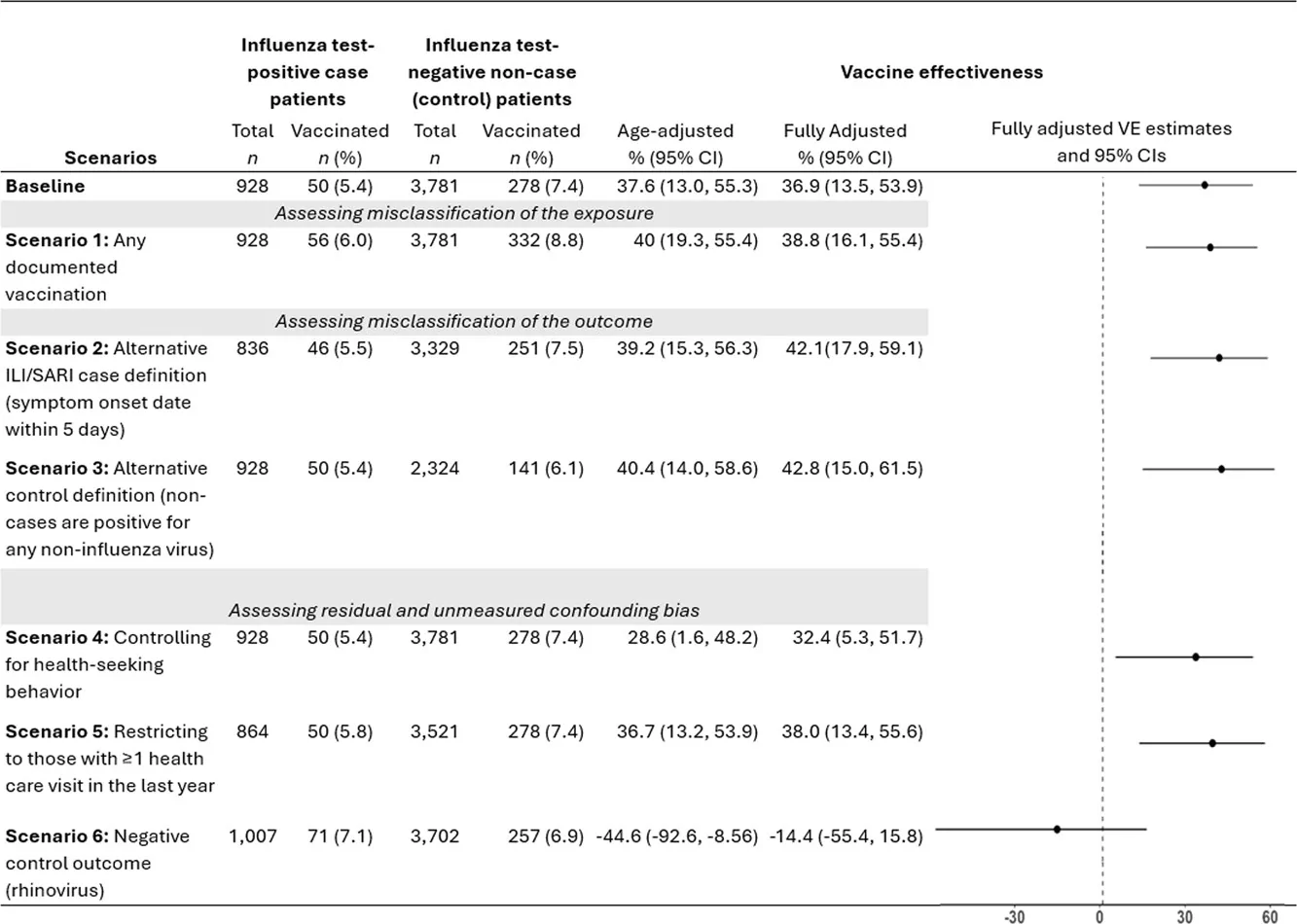
Influenza vaccine effectiveness calculated under each scenario, Thailand, June 2023-May 2024. Legend. Abbreviations**:** ILI = influenza-like illness, SARI = severe acute respiratory illness. Baseline model uses a logistic regression model adjusting for sex, age in years, sentinel site, COVID-19 vaccination, calendar week of symptom onset, and presence of at least 1 comorbidity. Scenario 1 uses a more intense method for classifying influenza vaccination status. Scenario 2 uses an alternative ILI and SARI case definition with symptom onset within 5 days rather than 10 days. Scenario 3 uses an alternative control definition where noncases must be positive for any noninfluenza virus rather than negative for influenza virus. Scenario 4 adds adjustment for prior health-seeking behavior. Scenario 5 restricts the analysis to patients with at least 1 health care visit in the year prior to enrollment in surveillance. Scenario 6 calculates influenza vaccine effectiveness against rhinovirus, used as a negative control outcome.

The baseline-adjusted vaccine effectiveness was 36.9% (95% CI, 13.5%-53.9%) (Figure 2). After including any available data source to determine influenza vaccination status (scenario 1), adjusted vaccine effectiveness was 38.8% (95% CI, 16.1%-55.4%). After changing the ILI/SARI case definition to symptom onset date within 5 days (scenario 2), adjusted vaccine effectiveness was 42.1% (95% CI, 17.9%-59.1%). Using positivity for any noninfluenza viruses except SARS-CoV-2 as the definition for noncases (scenario 3), adjusted vaccine effectiveness was 42.8% (95% CI, 15%-61.5%).

After controlling for number of health care visits in the previous 12 months as a proxy for health-seeking behavior (scenario 4), adjusted vaccine effectiveness was 32.4% (95% CI, 5.3%-51.7%). In a model restricted to those with at least 1 health care visit in the last 12 months (scenario 5), adjusted vaccine effectiveness was 38% (95% CI, 13.4%-55.6%). In the negative control model with rhinovirus as the outcome (scenario 6), adjusted vaccine effectiveness was –14.4% (95% CI, –55.4%-15.8%). Finally, after combining scenarios 1 and 2, adjusted vaccine effectiveness was 42.5% (95% CI, 20.0%-58.7%) and combining 1, 2, and 5, adjusted vaccine effectiveness was 42.7% (95% CI, 20.2%-58.9%).

## Discussion

We found little evidence that misclassification or confounding bias contributed appreciably to a change in influenza vaccine effectiveness estimates against medically attended influenza illness in Thailand. In 5 scenarios, vaccine effectiveness estimates were not meaningfully different from the baseline vaccine effectiveness estimate and had overlapping CIs that did not include the null value of 0. Influenza vaccine effectiveness estimates against medically attended rhinovirus in the same population produced a nonsignificant outcome, which was consistent with clinical expectations that influenza vaccine would not protect against rhinovirus. This finding suggested that estimates for influenza vaccine effectiveness were not driven by unmeasured confounding. After combining scenarios to account for multiple sources of bias, vaccine effectiveness estimates were consistent with other estimates and were not meaningfully different from the baseline estimate. Together, these findings suggest that the current test-negative design is a pragmatic approach in Thailand that produces reasonable vaccine effectiveness estimates.

Our analysis of misclassification of influenza vaccination status found that standard procedures missed 15% of vaccinations. Surveillance officers at each site often relied upon self-reported vaccine status if they were unable to verify the vaccination through another data source. Self-reported vaccination status can bias vaccine effectiveness estimates, but the magnitude depends on vaccine coverage and the degree of misclassification.^13^^,^^24^ In this analysis, the proportion of misclassified vaccination status among cases and noncases was relatively small. This suggests that even if there is some misclassification, in this setting where vaccine coverage is low, there may not be a large effect on vaccine effectiveness. If the degree of misclassification was greater, vaccine effectiveness might be underestimated or, in some cases, overestimated.^25^ In the long term, a national immunization registry that records vaccinations for patients of all ages from public and private sources would be beneficial for improved monitoring of vaccine performance measures, such as vaccine effectiveness.

In our analysis, we observed minimal variation in influenza vaccine effectiveness estimates when employing an alternative case definition for ILI and SARI. The rationale for adjusting the case definition to include symptom onset within 5 days was based on the premise that this time frame would enhance the sensitivity of influenza detection by rtPCR testing, as viral loads are typically higher shortly after onset. However, our findings suggest that this adjustment did not yield a large impact on vaccine effectiveness estimates. Additionally, while we noted a slight increase in vaccine effectiveness when redefining the noncase group to include any noninfluenza viral infections, except SARS-CoV-2, this change did not result in a meaningful difference compared to the broader category of any noninfluenza infections. Previous studies exploring alternative control groups, such as any noninfluenza-positive vs pan-negative controls, similarly reported no appreciable differences in vaccine effectiveness, as evidence by a systematic review and meta-analysis of 12 studies conducted prior to the COVID-19 pandemic.^22^ It is important to highlight that earlier vaccine effectiveness calculations utilizing various definitions of influenza, including ILI without laboratory confirmation and serological methods, have been associated with notable biases.^14^ The incorporation of laboratory confirmation and systematic selection of cases and noncases can mitigate selection bias, reinforcing the importance of rigorous case definitions in vaccine effectiveness assessments.^26^^-^^28^

Test-negative design studies are recognized for their ability to mitigate confounding related to health-seeking behavior.^12^ We observed notable differences in the number of health care visits in the previous year between the exposure and outcome groups, which served as a proxy for health-seeking behavior. However, when we controlled for the number of visits, the vaccine effectiveness estimates remained largely unchanged. This finding aligns with previous research indicating that while residual confounding by health-seeking behavior may persist, the differences in health-seeking behavior would likely need to be more extreme to significantly impact vaccine effectiveness estimates.^16^^,^^29^^-^^31^ Similarly, we did not see a difference in vaccine effectiveness in our analysis restricted to those with at least 1 health care visit in the last year, further supporting the notion that the influence of health-seeking behavior on vaccine effectiveness is mitigated with this study design. Additionally, a comparative study examining bias between the test-negative design and traditional case–control approaches found that the test-negative design exhibited less bias, provided that influenza vaccination is unrelated to noninfluenza respiratory illnesses.^31^ While modeling studies suggest that extreme health-seeking proclivity could affect vaccine effectiveness estimates, these models rely on observational data rather than direct assessments of real-world scenarios.^30^

It is important to note that the number of health care visits serves as an imperfect proxy for health-seeking behavior, as it may reflect overall health status. Health status exists on a spectrum and may not be fully controlled for by a binary comorbidity variable. The distribution of health care visits was highly right-skewed, with some patients having over 100 visits in the previous 12 months, indicating underlying medical conditions that necessitate frequent health care interactions. Interestingly, while other studies have posited that underlying medical conditions are positively associated with influenza case status as a risk factor for severe influenza,^13^ our findings suggested a negative association. This may be attributed to patients with underlying conditions presenting with fever and cough for reasons beyond influenza alone or a higher likelihood of vaccination among these patients. These observations raise questions about selection bias related to health-seeking behavior and underlying medical conditions, the answers to which would provide a clearer understanding of the impact of these factors on vaccine effectiveness. Evaluating other markers of health-seeking behavior, such as primary care visits and health screenings, could be included in future evaluations.^32^

To enhance data quality and validity of vaccine effectiveness calculations within Thailand’s sentinel influenza surveillance system, several operational and process improvements could be considered. First, automating the data retrieval of influenza vaccination records in the surveillance system could significantly reduce reliance on surveillance officers’ memory, thereby minimizing potential errors and inconsistencies. Implementing a more robust vaccination tracking system is essential; while an effective system was established during the COVID-19 pandemic, it was limited to COVID-19 vaccinations and is not sustainable in the postemergency phase. Expanding this framework to encompass influenza vaccinations would improve data accuracy and facilitate timely access to vaccination histories. Additionally, regular training for surveillance officers on data collection protocols and the importance of consistent and accurate reporting could further enhance the integrity of the surveillance system. By adopting these recommendations, Thailand could likely strengthen its influenza surveillance efforts and improve the reliability of vaccine effectiveness assessments in the future.

This study has several limitations. First, this study may be underpowered, resulting in wide CIs. This may have limited our ability to detect meaningful differences between vaccine effectiveness estimates and to precisely assess the degree of residual confounding. However, because the CIs largely overlap and do not cross the null, we believe our interpretation is reasonable. Additionally, the number of health care visits in the preceding 12 months serves as an imperfect measure of health-seeking behavior. Furthermore, children under 9 years of age receiving influenza vaccination for the first time should receive 2 doses of influenza vaccine but were classified as vaccinated after receiving at least 1 dose.^33^ Vaccine effectiveness might be higher among young children with 2 doses of influenza vaccine as 1 dose provides insufficient protection.^34^ Finally, there is no universally accepted “gold standard” for vaccination records; some sites accepted self-reported vaccination history while others did not, depending on subjective reliability of vaccination record sources. Privately purchased vaccine doses are particularly difficult to track because hospitals and providers do not submit vaccination records into the Krung Thai Digital Health Platform for reimbursement. This made it challenging to verify all self-reported vaccinations.

In conclusion, the findings of this study provide reassurance regarding the validity of Thailand’s application of the test-negative design for calculating influenza vaccine effectiveness. The results support the reliability of the estimates generated, which are crucial for informing public health strategies and vaccine strain selection. This study underscores the importance of robust surveillance methodologies in accurately assessing vaccine performance after they are licensed.

## Data Availability

The data underlying this article will be shared on reasonable request to the corresponding author.
